# GAS-STING: a classical DNA recognition pathways to tumor therapy

**DOI:** 10.3389/fimmu.2023.1200245

**Published:** 2023-10-18

**Authors:** Xinrui Wang, Meijia Lin, Liping Zhu, Zhoujie Ye

**Affiliations:** ^1^ National Health Commission (NHC), Key Laboratory of Technical Evaluation of Fertility Regulation for Non-Human Primate, Fujian Maternity and Child Health Hospital, Fuzhou, Fujian, China; ^2^ College of Clinical Medicine for Obstetrics & Gynecology and Pediatrics, Fujian Medical University, Fuzhou, Fujian, China; ^3^ Medical Research Center, Fujian Maternity and Child Health Hospital, Fuzhou, Fujian, China; ^4^ Department of Pathology, School of Basic Medical Sciences, Fujian Medical University, Fuzhou, China

**Keywords:** cGAS-STING pathway, DNA damage, type I IFNs, tumor immunotherapy, immune checkpoint inhibitor therapy

## Abstract

Cyclic GMP-AMP synthetase (cGAS), recognized as the primary DNA sensor within cells, possesses the capability to identify foreign DNA molecules along with free DNA fragments. This identification process facilitates the production of type I IFNs through the activator of the interferon gene (STING) which induces the phosphorylation of downstream transcription factors. This action characterizes the most archetypal biological functionality of the cGAS-STING pathway. When treated with anti-tumor agents, cells experience DNA damage that triggers activation of the cGAS-STING pathway, culminating in the expression of type I IFNs and associated downstream interferon-stimulated genes. cGAS-STING is one of the important innate immune pathways,the role of type I IFNs in the articulation between innate immunity and T-cell antitumour immunity.type I IFNs promote the recruitment and activation of inflammatory cells (including NK cells) at the tumor site.Type I IFNs also can promote the activation and maturation of dendritic cel(DC), improve the antigen presentation of CD4^+^T lymphocytes, and enhance the cross-presentation of CD8^+^T lymphocytes to upregulating anti-tumor responses. This review discussed the cGAS-STING signaling and its mechanism and biological function in traditional tumor therapy and immunotherapy.

## Introduction

Innate immunity serves as the host’s frontline defense against foreign microbial infections, playing a pivotal role in immune responses that clear viruses and bacteria. The detection of “foreign” DNA stands as one of the most fundamental mechanisms of host defense responses. Restriction endonucleases and Clustered Regularly Interspaced Short Palindromic Repeats (CRISPR) safeguard bacteria against the invasion of plasmid DNA and bacteriophages (1, 2). In mammalian cells, innate DNA receptors and associated intracellular signaling incite robust immune responses against extrinsic pathogenic microorganisms. The cGAS-STING signaling pathway acts as a key cellular effector, sensing and responding to foreign double-stranded DNA (dsDNA) in the cytoplasm, which stimulates the expression and secretion of type I IFNs and downstream interferon-stimulated genes (ISGs). This action contributes to establishing a natural immune defense response (3, 4).

Double-stranded DNA(dsDNA)which is independently of the DNA sequence can activate cGAS, Physical and chemical factors lead to genomic DNA damage, damaged mitochondria, genomic instability, virus and pathogen infection of cells,decreased activity of DNA exonuclease and other factors may lead to dsDNA in the cytoplasm. The dsDNA is identified by the DNA receptor cyclic GMP-AMP synthase (cGAS), thereby activating the cGAS-STING signaling pathway (5). This activated pathway encourages the production of various immune and inflammatory mediators, including type I IFNs and senescence-associated secretory phenotype (SASP) (6). As part of the innate immune system, the cGAS-STING pathway forms a critical surveillance system that responds to widespread tissue damage and pathogen invasions. Its dysregulation is linked with the pathogenesis of a broad range of human diseases, including infectious diseases, autoimmune diseases, cancer, and neurodegenerative diseases (7–11).

DNA damage acquired by precancerous cells during tumorigenesis and exposure to classical cancer treatments (radiotherapy or chemotherapy) induces DNA damage and micronucleus formation, thereby activating the cGAS-STING pathway and inducing the production of type I IFNs and other cytokines that enhance anti-tumor immunity (12, 13). Roger A. et al. reported that mitotic progression following dsDNA breakage leads to the formation of micronuclei, which are recognized by cGAS (14). The inhibition of mitotic progression or loss of pattern recognition in cancer cells prevents micronucleus formation, thereby suppressing the immune response (14). This review explores the self-regulatory mechanism of the cGAS-STING signaling pathway and expounds on its role in tumor immunotherapy, considering DNA sensing (cGAS senses dsDNA), the intracellular signaling cascade, and immune response activation.

## The cGAS-STING pathway

The cGAS-STING pathway is an evolutionarily conserved mechanism prevalent in several mammals, where it mediates immune responses against pathogens (15, 16). As a novel DNA sensor, cGAS recognizes extracellular dsDNA (originating from viral, bacterial, and plasmid DNA), along with cytoplasmic free DNA fragments derived from damaged DNA and mitochondrial DNA (17). Upon detecting cytoplasmic dsDNA, cGAS facilitates cGAMP production, employing ATP and GTP as substrates. The produced cGAMP binds to and activates STING on the endoplasmic reticulum (ER) membrane (18).

The C-terminal domain (CTD) of STING forms a V-shaped binding pocket that faces the cytoplasm. When inactive, STING is anchored to the ER in the form of butterfly-like dimers via several transmembrane domains (19). cGAMP binding triggers extensive conformational changes in the CTD, particularly in the V-shaped dimer, which adopts a closed conformation with a “lid” covering the cGAMP-binding site (20). This conformational change propels STING from the ER to the Golgi complex via the ER-Golgi intermediate compartment through an unknown mechanism (21, 22). The translocation of STING to the Golgi complex is vital for the activation of downstream signaling components and the regulation of type I IFNs transcription, which is a characteristic output signal of cGAS-STING activity.

**Figure 1:**
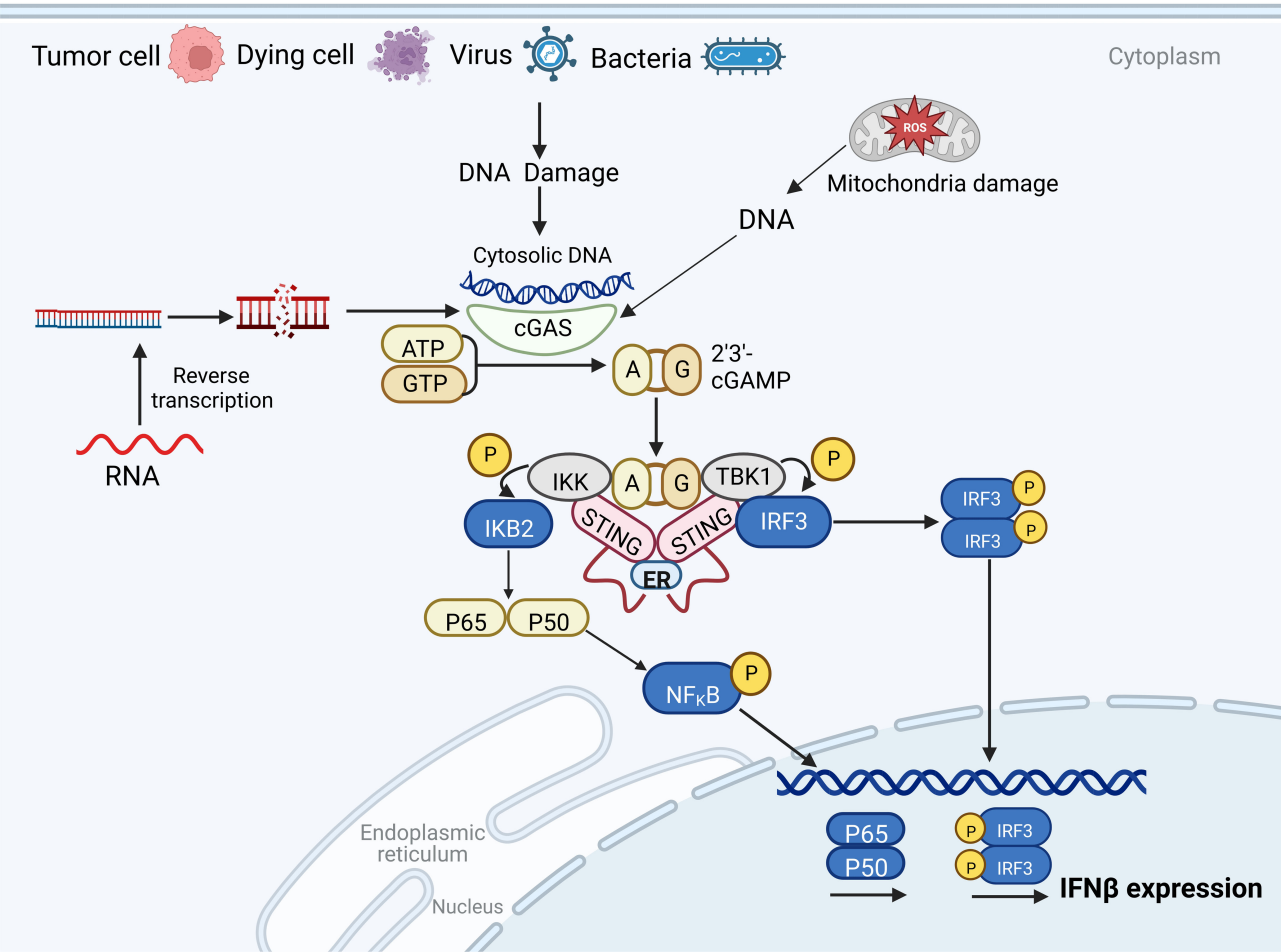
The cyclic GMP-AMP synthase (cGAS)-stimulator of interferon gene (STING) pathway. In host cells, cGAS recognizes cytoplasmic free DNA and catalyzes the formation of cyclic GMP-AMP (cGAMP), a second messenger. cGAMP activates STING to recruit TBK1, which phosphorylates IRF3, inducing the expression of type I IFNs. STING can also bind to nuclear factor κB (NF-κB) kinase inhibitor (IKK), which induces the transcriptional activation of NF-κB and regulates the expression and secretion of pro-inflammatory cytokines.

Within the Golgi complex, STING undergoes palmitoylation at two cysteine residues (Cys88 and Cys91) (23, 24), accounting for the necessity of STING’s translocation to the Golgi complex. Palmitoylation further enhances STING oligomerization and subsequent activation of TBK1. However, mere recruitment of TBK1 is insufficient to activate IRF3 (25). Instead, the phosphorylation of a conserved consensus motif (pLxIS; where p is a hydrophilic residue, and x represents any residue) in the STING CTD (S366), which is mediated by TBK1, proves crucial for the STING-IRF3 interaction (26, 27). TBK1 then phosphorylates IRF3, which subsequently dimerizes and translocates to the nucleus to initiate type I IFNs expression. Additionally, STING can interact with nuclear factor κB (NF-κB) kinase inhibitor (IKK), leading to the transcriptional activation of NF-κB and thereby controlling the expression and secretion of pro-inflammatory cytokines (28) (**Figure 1**).

## Post-translational modification of cGAS

Effective activation of cGAS within the innate immune pathway is crucial for countering exogenous pathogenic infections. However, excessive activation of cGAS might lead to immunological damage during responses to DNA viruses (29). The primary mechanism for cGAS activation involves its binding to dsDNA, but the regulation of the cGAS pathway also involves other mechanisms. For instance, post-translational modifications of the cGAS protein are pivotal in shielding the immune system against damage during responses to foreign pathogens (30, 31). These post-translational modifications primarily encompass phosphorylation, acetylation, and ubiquitination (29, 32, 33). In humans and mice, AKT can inhibit cGAS activity by phosphorylating residues S291 and S395 (34). Zusen Fan et al. demonstrated that mice lacking *Agbl5* or *Agbl6* were prone to DNA virus infection, and these proteins were necessary for the activation of type I IFNs in macrophages. AGBL5 catalyzes the hydrolysis of cGAS’s monoglutamate residue, whereas AGBL6 catalyzes the deglutamylation of cGAS’s polyglutamate side chains. Therefore, the glutamylation and deglutamylation of cGAS proteins jointly modulate immune responses (35). ZDHHC18-mediated palmitoylation can inhibit the activity of cGAS after its binding to DNA through a negative feedback mechanism, thereby negatively regulating the activation of downstream innate immune signaling pathways. This discovery sheds light on a novel and refined innate immune signaling pathway (36).

During cell division, the loss of the nuclear envelope allows cGAS to access genomic DNA. However, cGAS does not become hyperactivated to provoke an overly potent innate immune response. The research team led by Andrea Ablasser reported that the disappearance of the nuclear membrane barrier promotes BANF1’s competitive binding to DNA, which inhibits cGAS’s binding to genomic DNA, thereby preventing cGAS overactivation (37). Alongside various epigenetic modifications, cGAS activity is regulated by other genes. For instance, KU proteins (XRCC5 and XRCC6) directly interact with cGAS and positively regulate cGAS-mediated antiviral signaling (38). ZYG11B boosts natural immune responses by enhancing the binding of cGAS to exogenous free DNA (39). Further studies are imperative to unravel the regulatory mechanisms of the cGAS-STING pathway and to ascertain the role of this pathway in the progression of human diseases.

## Biological function of cGAMP

Cyclic GMP-AMP (cGAMP) is a second messenger synthesized from activated cGAS using ATP and GTP as substrates. In tumor cells, cGAMP produced following radiotherapy can function as extracellular immune mediators (40). The interstitial cGAMP, taken up by immune cells infiltrating the tumor microenvironment, activates the STING pathway in these cells, enhancing their cytotoxicity (41). Extracellular cGAMP can also activate STING in paracancerous cells through unknown mechanisms, further promoting natural killer (NK) cell responses (41). In addition to stimulating cells in an autocrine manner to trigger a signaling cascade response, cGAMP can be transferred to adjacent cells through gap junctions (42). Recent studies have identified that gap junctions, SLC19A1, P2X7R, LL37, and LRRC8 can facilitate the cell-to-cell or extracellular-to-intracellular transfer of cGAMP (43–47).

cGAMP can enhance the transcription of interferon and activate and recruit dendritic cells (DCs) (48). These DCs, along with macrophages and tumor cells, secrete chemokines like CXCL9 and CXCL10, which aid in recruiting circulating CXCR3-expressing CD8^+^ T cells to the tumor site to exert cytotoxic effects on tumor cells (49, 50). The recruited DCs enhance the recruitment of inflammatory cytokines and CD8^+^ T cells, promoting T-cell activation in the lymph nodes, initiating an immune response, and amplifying their cytotoxic effects against tumors (51, 52). Various factors mediating T cell homing play a crucial role in regulating tumor immunity (53). Interferons upregulate the expression of multiple chemokines, such as CXCL9 and CXCL10, promoting the migration and infiltration of cytotoxic T lymphocytes (CTLs) (54). Stress-induced immunogenic cell death triggers the release of cytokines like ATP, type I IFNs, IL-1β, and annexin A1, which regulate the function of both innate and adaptive immune cells in the tumor microenvironment (55).

Despite such a stringent immune regulatory response, some tumor cells still possess mechanisms for evading immune recognition by extracellular cGAMP. For instance, in the triple-negative breast cancer cell line MDA-MB-231, tumor cells can hydrolyze extracellular cGAMP via ENPP1, inhibiting immune cell activation. The adenosine produced following the hydrolysis of cGAMP further impedes antitumor immunity and fosters tumor metastasis (56). Nevertheless, this should not deter us from deepening our understanding of cancer and potentially discovering new therapeutic targets for innovative cancer treatments.

## Transcriptional and epigenetic regulation of the cGAS-STING pathway

Chronic activation of the STING pathway induced by chromosomal instability (CIN) can promote downstream changes in cell signal transduction, thereby inhibiting highly effective anti-tumor immunity, which is one of the reasons why the cGAS-STING pathway in tumor cells does not work “normally” (57). In addition, immune escape is achieved in many different types of tumors due to inactivation of cGAS/STING signaling due to loss-of-function mutations or epigenetic suppression (58, 59). The activity of cGAS is often diminished in tumor cells due to epigenetic modifications, a process mitigated by DNA methylation inhibitors (60). These immunomodulatory signals and genes include interferons, with DNA methyltransferase inhibitors (DNMTis) notably upregulating the expression of Interferon-stimulated genes (ISGs) in various cancer cells (61, 62). Zebularine, a DNMTi, not only inhibits tumor cell growth but also upregulates the expression of STING by inducing cytoplasmic DNA accumulation. Additionally, zebularine aids in the recruitment and activation of immune cells to exert anti-tumor effects, reducing tumor burden through the upregulation of type I IFNs, amplification of antiviral response, and other immunomodulatory mechanisms (60).

The research team led by David M. Ashley performed methylated DNA sequencing (Illumina) on tumor samples from patients with glioblastoma (GBM). This sequencing revealed that the CpG site cg16983159 on the STING promoter was hypermethylated, thereby inhibiting the expression of STING mRNA (59). The hypermethylation of cg16983159 was also observed in both healthy adult and fetal brains, suggesting that STING promoter methylation is conserved during brain development and tumorigenesis. Decitabine, a DNMTi, could suppress methylation at cg16983159 and upregulate STING expression in GBM cell lines, enabling the previously cGAMP-insensitive cells to respond to cGAMP stimulation and promoting the expression of type I IFNs (59, 63).

The downregulation of cGAS and STING genes through epigenetic modification is a shared characteristic among various tumor cells (64). Thus, it is essential to elucidate the regulatory mechanisms of the cGAS-STING signaling pathway in tumor cells. In STING-defective melanoma cell lines, restoration of STING signaling through demethylation was shown to upregulate HLA and promote the expression of MHC-I, which in turn enhances the recognition of tumor cells by killer T cells (58). This underscores the importance of epigenetic modifications in the cGAS-STING pathway, tumor immune evasion, and the development of tolerance to T cell immunotherapy. Furthermore, the role of DNA methylation in innate STING signal transduction dysfunction across various tumor cells is evident. The downregulation of cGAS-STING in diverse tumor cells may be closely linked to epigenetic modifications, offering a novel theoretical foundation for reactivating the cGAS-STING pathway to enhance anti-tumor immune responses.

## Apoptosis activates the cGAS-STING pathway

Apoptosis, an intrinsic process of cellular self-destruction, encompasses various biological changes such as nuclear membrane rupture, gene expression modulation, and genomic DNA breakage (65), each of which may activate the cGAS-STING signaling pathway. This mechanism serves to eliminate excessive, aged, and damaged cells, thereby preserving the body’s physiological functionality (66, 67). type I IFNs can induce tumor cell apoptosis via both the exogenous death receptor-mediated pathway and the endogenous mitochondrion-mediated pathway (68).

In breast cancer, paclitaxel can induce the activation of cGAS and release IFNβ and TNFa, thereby driving other cell apoptosis in a paracrine manner. Mechanically, these cytokines can induce the apoptosis regulator Noxa to increase the mitochondrial outermembrane permeabilization (MOMP) (69). At the same time, apoptotic cells may also be able to activate the cGAS-STING pathway in surrounding cells. In patients with advanced hepatocellular carcinoma (HCC), STAT3 knockdown promotes endoplasmic reticulum stress-induced apoptosis induced by sorafenib. Importantly, the DNA released by dying HCC cells stimulates the cGAS-STING signaling pathway in CD103^+^ DC and promotes type I IFNs production, thereby enhancing the anti-tumor function of CD8^+^ T and NK cells (70). Moreover, type I IFNs can activate CD8^+^ T cells and extend the overall survival of tumorigenic mouse models by impeding breast cancer cell metastasis (71).

Rautela et al. blocked endogenous type I IFNs and its downstream signaling pathways in wild-type mice by knocking out the type I IFNs receptor (creating C57BL/6I*^fnar1−/−^* mice). Consequently, the cytotoxicity of NK cells against tumor cells was markedly attenuated in these mice, leading to an accelerated bone metastasis of breast cancer (41). This underlines the crucial role of type I IFNs in lymphocyte activation, strengthening tumor immune surveillance, and inhibiting tumor metastasis.

## The cGAS-STING pathway and cellular senescence

Cellular senescence, a process marked by the gradual decline in a cell’s proliferation, differentiation capacity, and physiological function over time, can be triggered by various stressors such as exogenous stress, drug stimulation, and genomic instability (72, 73). These stressors induce a state of replicative cycle arrest, preventing the transmission of damaged DNA to subsequent generations while staving off potential cellular deterioration (73). Beyond inducing cycle arrest, senescent cells also secrete a plethora of inflammatory cytokines, chemokines, growth factors, and proteases, a phenomenon referred to as the SASP (74). Chemokines and proinflammatory factors, products of SASP, can activate and recruit lymphocytes to eliminate cells harboring abnormal, damaged DNA (74).

Cellular senescence can promote micronucleus formation, thereby activating cGAS (75, 76). Abnormal cytoplasmic DNA (CCFs), released due to genomic instability caused by senescence, is recognized by cGAS, triggering the cGAS-STING pathway. This induces pro-inflammatory factors to clear senescent cells and prevent their transformation into tumor cells (77). Consequently, cGAS-STING-mediated DNA damage perception is essential for SASP. In cells where cGAS or STING is deleted, the senescence process slows in response to various stresses (78). The absence of cGAS expedites the spontaneous immortalization of mouse embryonic fibroblasts (78) and eliminates SASP induced by spontaneous immortalization or DNA damaging agents, including radiation and etoposide (79). In patients with human lung adenocarcinoma, low expression of cGAS is associated with poorer survival outcomes (70).

Studies have shown that with age, the abundance of autophagy associated proteins gradually declines and less cargo is delivered to lysosomes, suggesting that impaired autophagy is a major feature of aging in the body (80). Recent studies have shown that the activation of cGAS-STING pathway is closely related to autophagy. After DNA stimulation, STING protein was co-located with autophagy marker LC3 (81). cGAS and Beclin-1 promoted autophagy through interaction. This interaction inhibits cGAS catalytic activity, thereby negatively modulating the cGAS-STING pathway to avert excessive immune activation of this pathway (82). Furthermore, STING has been reported to activate autophagy independently of TBK1 and interferon induction, thus preventing tumorigenesis (21). These findings suggest that SASP induction via the cGAS-STING pathway may avert cancer by instigating senescence processes that enhance lymphocyte-mediated clearance of abnormal cells through proliferation.

## cGAS-STING and chromosome instability in tumor cells

In DNA mismatch repair-deficient (MMRd) tumor cells, DNA leakage into the cytoplasm occurs (83). Upon DNA damage and spillage, cGAS recognizes and activates the expression of interferon and other immune factors, which, in conjunction with anti-inflammatory cytokine release, can recruit lymphocyte infiltration to effectuate tumor cell eradication (84). Given that persistent chronic inflammation in chromosomally unstable tumors can foster tumor development, it has been discovered that cGAS-STING signaling inactivation can selectively inhibit the survival of chromosomally unstable (CIN) triple-negative breast cancer cells (85). CIN instigates IL-6-STAT3 mediated signaling via the cGAS-STING pathway and the noncanonical NF-kB pathway, thus promoting tumor cell growth and metastasis. When DNA damage is detected in cells, cGAS can be translocated to the nucleus and recruited to DNA damage sites. By interfering with the formation of the PARP1/Timeless complex, cGAS inhibits DNA homologous recombination repair, reducing genomic stability and fostering tumorigenesis (86). These studies have shown that the cGAS-STING pathway exerts multifaceted effects on tumors, with different tumor types influencing distinct mechanisms of cGAS-STING action. Hence, comprehensive exploration of these diverse mechanisms offers promising avenues for the development of targeted therapeutics and may significantly improve the prognosis of patients with various types of cancer.

## The cGAS-STING and radiotherapy of cancer

Radiation therapy, an essential cancer treatment method alongside surgery and chemotherapy, employs ionizing radiation to instigate intracellular DNA double-strand breaks, thus leading to cell death (87). In addition to directly killing tumor cells, radiotherapy exerts distant effects to reduce tumor size (87). Throughout this process, weakened and damaged tumor cells release immune-stimulating proteins and cancer antigens. Macrophages and dendritic cells (DCs) present these antigens, thereby activating cytotoxic T-lymphocytes (CTLs) which seek out and assault cancer cells unexposed to radiation (88). This suggests that DNA damage-induced cell death is not the sole anticancer mechanism at play in radiation therapy.

Radiation induces an increase in the number of intracellular micronucleus, and micronucleus rupture leads to double-stranded DNA accumulation, which can be sensed by the cGAS-STING pathway (89). This mechanism links genomic instability to innate immune responses.A wealth of prior research indicates that immune cells, especially CD8^+^ T cells, are pivotal in determining the therapeutic efficacy of radiotherapy (90). Moreover, Radiotherapy induced the activation of intracellular cGAS-STING pathway and upregulated the expression of type I IFNs and other cytokines.the activation of type I IFNs at tumor sites and type I IFNs receptor activation on immune cells, both induced by radiation, are crucial to the efficacy of radiotherapy (91). Various studies have illustrated that radiation-induced DNA damage and subsequent cell death can trigger antitumor immune responses via the activation of the cGAS-STING pathway (92, 93).

However, while high radiation doses during radiotherapy serve to exterminate tumor cells, they can also inadvertently inflict DNA damage on immune cells, a common side effect of radiotherapy (94). At radiation doses of 12–18 Gy, the DNA exonuclease TREX1 present in tumor cells can degrade the DNA that has accumulated within the nucleus, thereby reducing immunogenicity. This action of TREX1 inhibits the cGAS-STING-mediated expression of type I IFNs, in turn attenuating the cytotoxic effects of CD8^+^ T cells on tumor cells (95, 96). Clinical studies have demonstrated that combining radiotherapy with immune checkpoint inhibitors can enhance therapeutic efficacy by upregulating PD-L1 expression and activating the cGAS-STING pathway (96, 97). To illuminate the immunomodulatory mechanism of tumor radiotherapy and evaluate its potential for combination with other forms of immunotherapy, clinical trials involving the concomitant use of immune checkpoint inhibitor therapy and radiotherapy are currently in progress. Such trials are poised to provide novel insights into alternative treatment options for tumors.

## The cGAS-STING and chemotherapy

Chemotherapy, as a principal therapeutic strategy for cancer, was once thought to only exert cytotoxic effects directly. However, emerging studies suggest that chemotherapy can also activate anti-tumor immunity (98). Chemotherapeutic drugs can induce cellular DNA damage, resulting in the upregulation of downstream chemokines such as CXCL10 and CXCL9 through the cGAS-STING pathway. This consequently augments the number of dendritic cells (DCs) and circulating tumor antigen-specific CD8^+^ T cells within the tumor microenvironment (99). Additionally, chemotherapeutic drugs can synergize with immune checkpoint inhibitors, enhancing survival and establishing enduring anti-tumor immune memory (64). It is noteworthy that micronuclei formation during chemotherapy can activate cGAS, thereby triggering anti-tumor immune responses (100). These insights have opened new frontiers in anti-cancer drug development, showing that chemotherapeutic drugs exert immunostimulatory effects in addition to their cytotoxic capabilities.

Specific chemotherapy drugs, such as cyclophosphamide, can promote the exhaustion of regulatory T cells (Tregs) and restore the effector function of T cells and natural killer (NK) cells (101). Cisplatin has the ability to upregulate MHC-I expression, thereby directly enhancing T cell function (102). Doxorubicin is strongly linked to the inhibition of myeloid-derived suppressor cells (MDSCs), the upregulation of type I IFNs, and the induction of immunogenic cell death (103). Importantly, low-dose chemotherapy or radiotherapy can stimulate anti-tumor immune responses. Prior studies have demonstrated that radiation can induce type I IFNs production via the cGAS-STING signaling pathway, thereby enhancing T cell priming.

**Figure 2:**
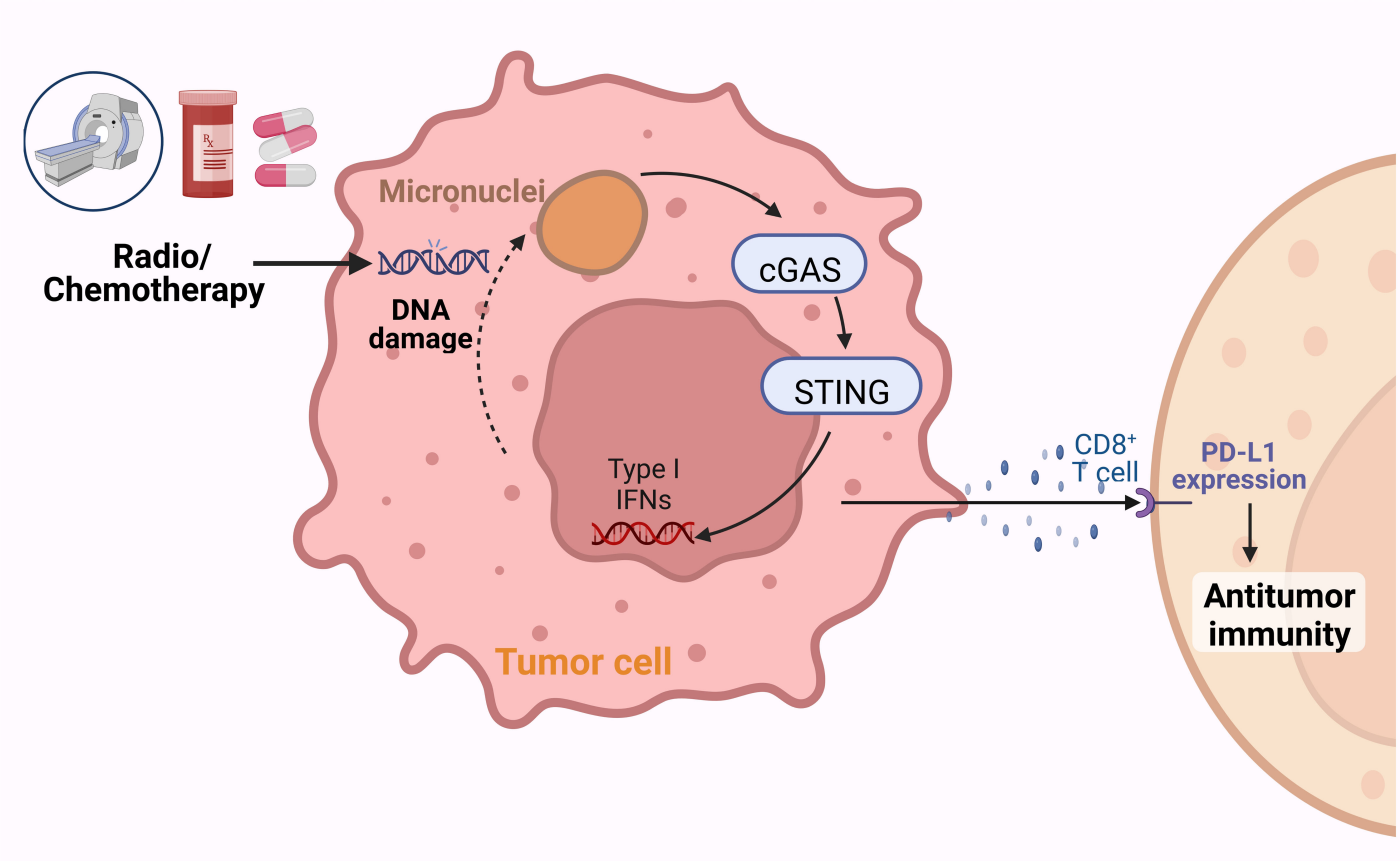
Radiotherapy and chemotherapy enhance the cytotoxic effects of lymphocytes against tumors. Chemotherapy and radiotherapy can induce DNA damage and consequently promote tumor cell death. The damaged DNA can activate the cyclic GMP-AMP synthase (cGAS)-stimulator of interferon gene (STING) signaling pathway, inducing the expression of type I IFNs and promoting the infiltration and cytotoxic effects of CD8^+^ T cells.

In the context of BRCA-deficient triple-negative breast cancer models, the therapeutic efficacy of PARP inhibitors hinges on the recruitment of CD8^+^ T cells to the tumor through the activation of the STING pathway. This recruitment results in an upregulation of PD-L1 expression on cancer cells upon treatment with PARP inhibitors (104). Consequently, PARP inhibitors can increase tumor sensitivity to anti-PD-L1 immunotherapy. PARP inhibitors also have the capacity to activate the cGAS-STING pathway in immune cells, thereby inducing anti-tumor immunity by effectively exterminating cancer cells and promoting the release of tumor-derived DNA (105). Moreover, when tumor cells sustain DNA damage response pathways or lack cGAS, the combination of STING agonists and PARP inhibitors can be utilized to further bolster anti-tumor immunity, effectively broadening the applicability of PARP inhibitors (**Figure 2**).

## cGAS-STING pathway and immune checkpoint inhibitor therapy

**Figure 3:**
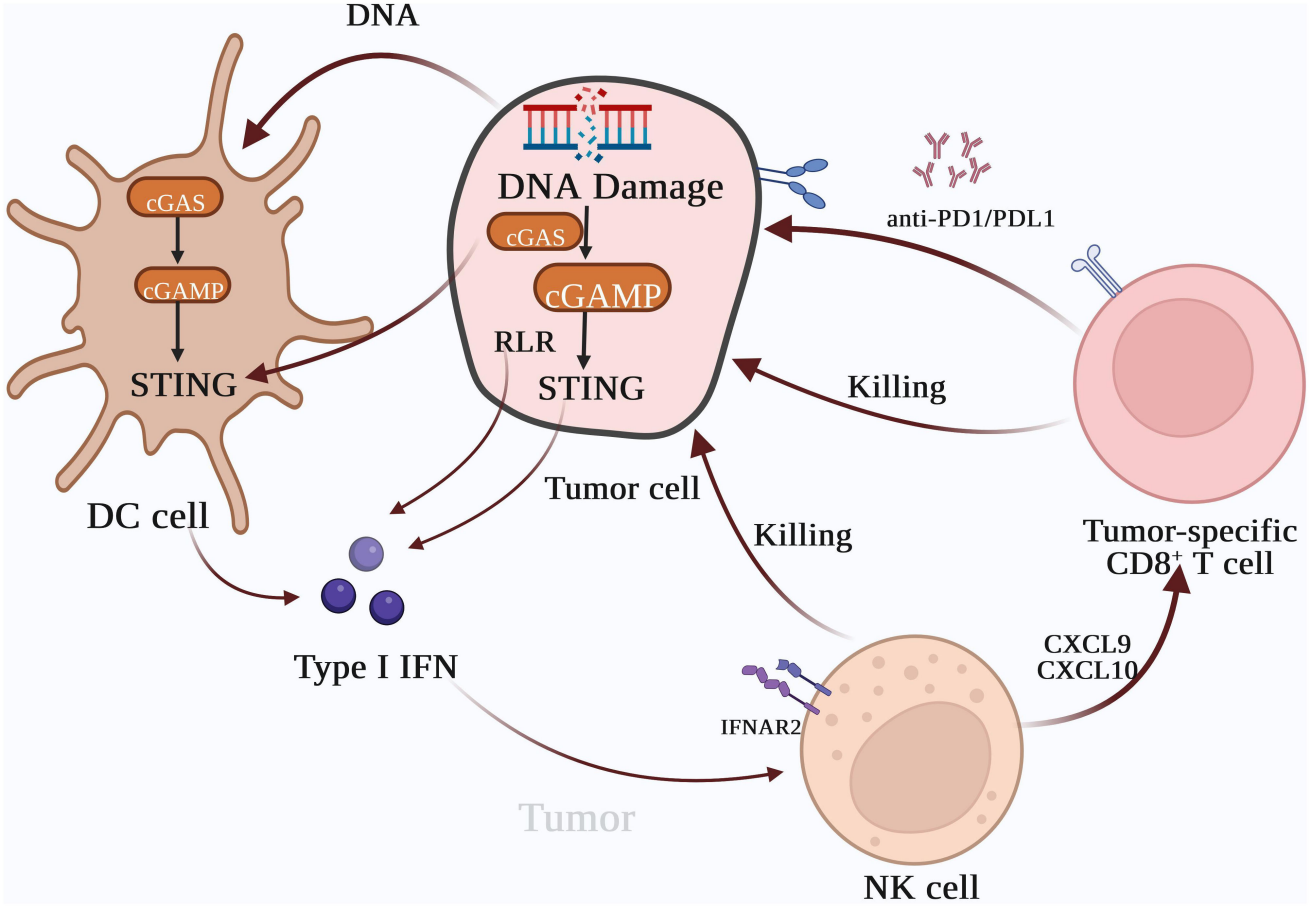
Type I IFNs are the bridge between innate immunity and T cell-based anti-tumor immunotherapy. The activation of the cyclic GMP-AMP synthase (cGAS)-stimulator of interferon gene (STING) pathway can induce the expression of Type I IFNs, which can recruit inflammatory cells and natural killer cells to tumor tissues. Chemokines, such as CXCL10 and CXCL9 can also enhance the cytotoxic effect of tumor-specific CD8^+^ T cells on tumors. Type I IFNs enhance lymphocyte-mediated tumor immune responses and promote the cytotoxic effects of lymphocytes in combination with tumor immune checkpoint inhibitors, which inhibit the interaction between tumor cells and lymphocytes, resulting in immune escape.

The emergence of cGAS-STING mediated cancer immune surveillance coupled with the application of immunotherapy have inaugurated a new epoch in cancer treatment. Immunotherapy, by bolstering the immune system’s capacity to recognize and eradicate specific cancer cells, provides a novel modality to combat cancer (106). Both radiotherapy and chemotherapy can facilitate the release of free DNA from damaged cells and instigate anti-tumor immunity through the activation of the cGAS-STING signaling pathway, an integral part of cancer treatment (4, 86, 107) (**Figure 3**). Activation of STING can induce downstream gene products and improve tumor immune microenvironment, the downstream type I IFNs can enhances the cytotoxic activity of immune cells such as macrophages, dendritic cells (DCs), B cells, and T cells against tumor cells (108). Numerous studies have shown that activation of STING and anti-PD-1/PD-L1 combination therapy show synergistic tumor growth inhibition in animal models such as breast cancer and melanoma (109, 110). In addition, a variety of STING agonists have entered the stage of clinical research, and activation of STING pathway as a promising cancer treatment will be further elaborated in this paper.

Moreover, type I IFNs can trigger CD8^+^ T cells and NK cells and escalate tumor antigen levels (41). Activated DCs present tumor surface antigens to T lymphocytes, bolstering the cytotoxic impact of CD8^+^ T cells on tumor cells. Within the immune system, regulatory T (Treg) cells contribute to anergy and immune suppression (111). Treg cells can impede lymphocyte recognition of tumor cells through various mechanisms. An upsurge in Treg cells can facilitate tumor immune escape, while activation of FOXP3^+^CD25^+^CD4^+^ Treg cells can markedly dampen tumor immune response (112). Type I IFNs can curb the proliferation of Treg cells, reduce the number of myeloid-derived suppressor cells (MDSCs), and inhibit their suppressive effects on immune activation (113).

Endogenous cGAS-STING signals can promote the differentiation of naive T cells into stem cell-like memory T cell (Tscm) subsets, which display traits of both memory cells and stem cells. The phenotype of Tscms in cancer patients is characterized as CCR7^+^CD62L^+^CD45RO^−^CD95^+^ (114, 115). Activation of the endogenous cGAS-STING pathway can amplify the cytotoxic effects of lymphocytes on tumors. Furthermore, the endogenous cGAS-STING pathway can significantly enhance the therapeutic efficacy of human chimeric antigen receptor T cells in solid tumors (64).

## Activation of innate immunity in tumor therapy

Immune checkpoint inhibitor therapy represents an innovative treatment avenue within oncology (116). The utilization of different antibodies that target immune checkpoints, such as PD-1, PD-L1, and CTLA-4, has produced beneficial outcomes for cancer patients (117, 118). This therapy enhances the immune responses of T cells against tumors characterized by low immunogenicity. The absence of tumor antigens, activated T cells, and lymphocyte homing, coupled with defective antigen-presenting cells (108), results in diminished T cell immune responses, thereby reducing the efficacy of immune checkpoint therapy (119).

A promising strategy to enhance the efficacy of tumor immunotherapy involves bolstering the response rate of patients by increasing T cell infiltration via tumor immune checkpoint inhibitors. Mouse tumor-bearing models lacking cGAS or STING were more prone to tumor growth, and in models (120, 121), the deficiency of cGAS and STING impaired the response of mouse models to immune checkpoint inhibitor therapy (122, 123). Tumor cells are absorbed by dendritic cells and macrophages, and tumor DNA may enter the cytoplasm from the phagosomes of antigen-presenting cells to activate the cGAS-STING pathway.Activation of cGAS by cancer cell DNA leads to the production of type I IFNs and chemokines, as well as the upregulation of co-stimulatory molecules such as CD80 and CD86, which jointly stimulate the proliferation and recruitment of tumor-specific T cells into the tumor,thus enhancing the anti-tumor immune response (124).

## Application of STING agonists in cancer research

The administration of STING agonists, either independently or in combination with immune checkpoint inhibitors, presents a novel therapeutic strategy for cancer. Several STING agonists are currently under clinical investigation (**Table 1**). STING agonists, which include cyclic dinucleotides (CDNs) and their derivatives (125), non-CDN small-molecule agonists (126), and antibody-drug conjugates, represent a groundbreaking class of small-molecule intrinsic immune agonists (127). The low molecular weight of STING agonists enables high tumor distribution through systemic administration. Furthermore, STING agonists have the potential to overcome the limitations of immune checkpoint inhibitors and exert robust anti-tumor effects on “cold tumors” by facilitating the initiation and recruitment of tumor-specific CD8^+^ T cells. The synergistic effect of STING agonists and immune checkpoint inhibitors can be beneficial for patients with cancer.

**Table 1:** Clinical trials on stimulator of interferon gene (STING) agonists.

Types of drugs	Sponsor	Drug administration	NCT number	Condition or disease	Status	Route of Delivery
Bacterial Vectors	Synlogic	SYNB1891	NCT04167137	Metastatic Solid Neoplasm; Lymphoma	I (Recruiting)	Intratumoral
CDN compounds	Chinook Therapeutics	ADU-S100	NCT03937141	Metastatic Head and Neck Cancer; Recurrent Head and Neck Cancer	II (Terminated)	Intratumoral
	Chinook Therapeutics		NCT02675439	Advanced/Metastatic Solid Tumors or Lymphomas	I (Terminated)	Intratumoral
	Novartis Pharmaceuticals		NCT03172936	Solid Tumors and Lymphomas	Ib (Terminated)	Intratumoral
	Bristol-Myers Squibb	BMS-986301	NCT03956680	Advanced Solid Cancers	I (Active, not recruiting)	Intramuscular
	Merck Sharp & Dohme LLC	MK-1454	NCT04220866	Head and Neck Squamous Cell Carcinoma	II (Completed)	Intratumoral
	Merck Sharp & Dohme LLC		NCT03010176	Solid Tumors; Lymphoma	I (Completed)	Intratumoral
	F-star Therapeutics	SB11285	NCT04096638	Melanoma; Head and Neck Squamous Cell Carcinoma; Solid Tumor	I (Recruiting)	Intravenous
	ImmuneSensor Therapeutics Inc	IMSA-101	NCT04020185	Solid Tumor, Adult	I (Recruiting)	Intratumoral
	Merck Sharp & Dohme LLC	MK-2118	NCT03249792	Solid Tumor; Lymphoma	I (Completed)	Intratumoral
	Neoplasms	BI-1387446	NCT04147234	Boehringer Ingelheim	I (Active, not recruiting)	Intratumoral
	Takeda	TAK-676	NCT04879849	Carcinoma of non-small cell lung; Triple Negative Breast Neoplasms; Squamous Cell Carcinoma of Head and Neck	Recruiting	Intravenous
	Eisai Inc	E-7766	NCT04144140	Lymphoma; Advanced Solid Tumors	I (Completed)	Intratumoral
	Shanghai De Novo Pharmatech	dn-015089	CTR20212462 (china)	Solid tumour	I (Recruiting)	Subcutaneous,Intratumoral
Non-CDN small molecules	Eisai	E7766	NCT04144140	Lymphoma; Advanced Solid Tumors	I (Completed)	Intratumoral
	Eisai		NCT04109092	Urinary Bladder Neoplasms	I ( Withdrawn)	Intravesical
	Merck Sharp & Dohme LLC	MK-2118	NCT03249792	Solid Tumor; Lymphoma	I (Completed)	Intratumoral
	Stingthera	SNX281	NCT04609579	Advanced Solid Tumor	I (Recruiting)	Intravenous
	Takeda	TAK676	NCT04879849	Non-small-cell lung Cancer; Triple-negative Breast Cancer; Squamous-cell carcinoma of the head and neck	I (Recruiting)	Intravenous
	GlaxoSmithKline	TTI-10001	NCT05424380	Acute Myeloid Leukemia	I (Recruiting)	Intravenous
	HitGen Inc.	GSK-3745417	NCT04998422	Advanced Solid Neoplasm	I (Recruiting)	Intravenous
	Sichuan Kelun-Biotech Biopharmaceutical	HG-381	CTR20221772 (china)	Solid tumour	I (Recruiting)	Intratumoral
Nanovaccines	Mersana Therapeutics	ONM-500	NCT05514717	Advanced/recurrent Solid tumors that express HER2	I (Suspended)	Intravenous
Antibody-drug conjugate	Mersana Therapeutics	XMT-2056	NCT05514717	Advanced/recurrent Solid tumors that express HER2	I (Suspended)	Intravenous
	Takeda	TAK-500	NCT05070247	Locally advanced or metastatic solid tumors	I (Recruiting)	Intravenous
	Codiak BioSciences	CDK-002	NCT04592484	Advanced Solid Neoplasm	I/II (Completed)	Intratumoral

CDNs, as the first-generation agonist of STING, are small molecule ligands that can bind to STING proteins and activate immune pathways for tumor immunotherapy (128, 129). The GL261 and CT-2A cell lines were employed to construct glioblastoma (BGM) animal models in mouse brains. ADU-s100, a STING agonist, and as a small molecule CDNs drug, significantly increased both survival and long-term survival with immune memory in GL261 models, suggesting the therapeutic potential of STING in BGM (130). When administered systematically, CDNs tend to induce the secretion of inflammatory cytokines in both tumor and normal tissues. Given their short half-life and poor metabolic stability, CDNs-based STING activators are primarily administered via direct intratumoral injection in clinical trials. However, intratumoral injection also limits their application in a broader range of tumor therapies (131, 132).

MSA-2, reported by Merck, is an oral non-nucleotide STING agonist that activates STING in the form of a non-covalent dimer to appear in a “closed” structure. MSA-2 demonstrated favorable antitumor effects in mouse models of colorectal cancer and was able to confer long-term anti-tumor immunity (133). In addition, it was found that MSA-2 and TGF-β/PD-L1 bispecific antibody YM101 can activate the innate immune system and overcome immunotherapy resistance (134). SR717, a non-nucleotide small molecule STING agonist obtained by large-scale drug screening targeting the cGAS-STING pathway in THP1 cells, also showed notable anti-tumor activity in mouse tumor models. SR717 was able to promote the activation of lymphocytes and cross-presentation of antigens (135).

Antibody-drug conjugate (ADC), which leverages the specificity of antibodies to couple STING agonists to tumor-specific antibodies, concentrates the payload on the tumor and is thus capable of reducing unnecessary exposure to normal tissue cells. Zhijian J. Chen et al. conjugated cGAMP analogue IMSA172 into mu-αEGFR antibody, and the ADC drug exhibited a potent anti-tumor effect in a melanoma mouse model. When further combined with an anti-PD-L1 antibody, an exceptional anti-tumor effect was observed (127). The utilization of bacteria to deliver STING agonists to tumor interiors is also under active investigation (136, 137).

As a natural STING agonist, manganese can lead to the release in organelle when cells are infected by virus, which activates the cGAS-STING pathway of cells, greatly improves its response to cytoplasmic DNA, and even enables it to be activated at the level of DNA that is originally not active (138). As STING agonist, Mn^2+^ can up-regulate antigen-presenting cells’ ability to present tumor antigens through cGAS-STING, and promote tumor tissue invasion and specific killing ability of CD8^+^T cells. When Mn^2+^ is combined with PD-1 antibody, the tumor therapeutic effect of PD-1 antibody can be significantly enhanced in a variety of tumor models (139), and the synergistic effect of manganese and TGF-β/PD-L1 bispecific antibody YM101 can also show significant tumor inhibition (140).

Pharmacological activation of STING has been demonstrated to be an effective cancer immunotherapy approach in a variety of preclinical models (141). Importantly, overactivation of STING results in the sustained production of cytokines, leading to uncontrolled inflammation and cytokine storms, tissue toxicity, autoimmunity, and an inflammatory tumor microenvironment that promotes tumor growth (142, 143). As research continues to advance, the development of systemic STING agonist administration strategies and the targeting of drugs under systemic administration mode bear significant research implications.

## Conclusions and future directions

Zhijian J. Chen and other researchers have underscored the critical role of the cGAS-STING pathway in immune responses to a broad spectrum of DNA pathogens and retroviruses (3, 5). This review presents the cGAS-STING signaling pathway, examines its primary regulatory role and function in anti-tumor responses, and explores tumor immunotherapy strategies that could pave the way for future research. Beyond inducing cell death, radiotherapy, chemotherapy, and other therapeutic interventions can provoke DNA damage in tumor cells, leading to the formation of micronuclei as a result of reactive oxygen species accumulation, radiation stress, chromosomal segregation errors, and replication pressure (4, 144). Micronuclei disintegration can expose genomic DNA to the cytoplasm, which can then be recognized and seized by cGAS. type I IFNs, secreted via the cGAS-STING pathway, augment antigen presentation through dendritic cells and amplify the cytotoxic effects of T lymphocytes.

The cGAS-STING pathway explains the immunomodulatory function of classical cancer therapy,The cGAS-STING pathway enhances the anti-tumor immune response by detecting the formation of micronucleus induced by DNA damage and chromatin fragments present in the cytoplasm. cGAS-STING pathway connects the cytotoxic effect and immune response into a new tumor treatment model. Classical cancer therapy combined with immune checkpoint inhibitor therapy showed synergistic antitumor effects. Activation of cGAS-STING pathway further promotes the infiltration of lymphocytes in the tumor immune microenvironment to enhance anti-tumor immunity (144, 145).

The upregulation of the cGAS-STING signaling pathway to augment the cytotoxic activity of lymphocytes presents a novel strategy for tumor immune checkpoint inhibitor therapy. Inhibiting the interaction of PD-1/PD-L1, CTLA4, and other immune checkpoints on tumor cells and lymphocytes, coupled with activating the cGAS-STING signaling pathway and its downstream type I IFNs signaling pathway using STING agonists or other pathways, opens a new approach to increase the sensitivity of tumors to immune checkpoint inhibitors.

In conclusion, our understanding of the cGAS-STING pathway has been greatly enriched, extending beyond its function as a cytoplasmic nucleic acid sensor, and highlighting the relationship between the DNA receptor cGAS and immune responses against pathogens and tumors (146). However, persistent activation of cGAS-STING led to a reduction in type I IFNs production and an increase in atypical NF-κB signaling in tumor cells with chromosomal instability (147). Chronic activation of the cGAS-STING pathway can promote tumor formation and metastasis by inducing inflammation. Recent findings suggest that STING signaling activation fosters the expansion of a subset of B regulatory cells with immunosuppressive functions (148). Therefore, a thorough understanding of the oncogenic or tumor-suppressive effects of cGAS-STING could assist in stratifying cancer patients for treatment. The cGAS-STING signaling pathway represents a promising immunotherapeutic target for inflammatory diseases and cancer. This review provides a valuable reference for the development of innovative clinical treatment strategies for cancer.

## Author contributions

ZY conceptualized the review and revised the manuscript; XW, ML, and LZ collected literature; XW prepared the first draft. All authors contributed to the article and approved the submitted version.
